# Two of Them Do It Better: Novel Serum Biomarkers Improve Autoimmune Hepatitis Diagnosis

**DOI:** 10.1371/journal.pone.0137927

**Published:** 2015-09-16

**Authors:** Saveria Mazzara, Antonia Sinisi, Angela Cardaci, Riccardo Lorenzo Rossi, Luigi Muratori, Sergio Abrignani, Mauro Bombaci

**Affiliations:** 1 Istituto Nazionale Genetica Molecolare “Romeo ed Enrica Invernizzi”, Milan, Italy; 2 DISSCO, Department of Clinical Sciences and Community Health, University of Milan, Milan, Italy; 3 Center for the Study and Treatment of Autoimmune Diseases of the Liver and Biliary System, Policlinico di Sant’Orsola, Department of Medical and Surgical Sciences (DIMEC), Alma Mater Studiorum, University of Bologna, Bologna, Italy; Hospital Israelita Albert Einstein, BRAZIL

## Abstract

**Background:**

Autoimmune hepatitis (AIH) is a chronic liver disease of unknown aetiology and characterized by continuing hepatocellular inflammation and necrosis. Autoantibodies represent accessible markers to measure the adaptive immune responses in the clinical investigation. Protein microarrays have become an important tool to discriminate the disease state from control groups, even though there is no agreed-upon standard to analyze the results.

**Results:**

In the present study 15 sera of patients with AIH and 78 healthy donors (HD) have been tested against 1626 proteins by an in house-developed array. Using a Partial Least Squares Discriminant Analysis (PLS-DA) the resulting data interpretation led to the identification of both new and previously identified proteins. Two new proteins AHPA9419 and Chondroadherin precursor (UNQ9419 and CHAD, respectively), and previously identified candidates as well, have been confirmed in a validation phase by DELFIA assay using a new cohort of AIH patients. A receiver operating characteristic analysis was used for the evaluation of biomarker candidates. The sensitivity of each autoantigen in AIH ranged from 65 to 88%; moreover, when the combination of the two new autoantigens was analyzed, the sensitivity increased to 95%.

**Conclusions:**

Our findings demonstrate that the detection of autoantibodies against the two autoantigens could improve the performance in discriminating AIH patients from control classes and in combination with previously identified autoantigens and they could be used in diagnostic/prognostic markers.

## Introduction

Autoimmune hepatitis is a complex disease and the diagnosis requires the exclusion of other conditions and the presence of characteristic features such as specific autoantibodies. Presently, these autoantibodies have relatively low sensitivity and specificity and are identified via immunostaining of cells or tissues. Indeed, there are problems such as standardization and interpretation of the immunostaining patterns [1]. To overcome these methodological problems, the International Autoimmune Hepatitis Group established an international committee to define guidelines, develop procedures and reference standards for more reliable testing [2, 3]. Moreover, serological overlap is frequently observed between AIH and other non-autoimmune liver diseases such as chronic viral hepatitis [4]. Therefore, new and highly specific markers represent an unmet medical need for a more accurate diagnosis and classification of AIH. Autoantigens identification represents a great contribution in early diagnosis and prognosis in autoimmune diseases. The use of human protein microarrays has become one of the most invaluable tool in the field of large-scale and high-throughput biology [5], and their use in basic research, diagnostics and drug discovery has emerged as a great promise of medicine [6]. An interesting application of this technology is the identification of a serodiagnostic antigens ensemble whose expression profiles can effectively unveil discriminating patterns providing the classification of healthy and disease samples [7–9].

However, to date, the data analysis of protein microarrays to extrapolate biologically interpretable results suffers from many issues that are still subject to debate and there is a compelling necessity for bioinformatics strategies in which the identification of novel disease biomarkers is performed automatically [10]. In particular, in protein microarrays, the achievement of biomarkers discovery depends on powerful antigen selection methods that can deal with a low sample size and a high number of features [11].

Literature provides a wide spectrum of data mining methods to overcome the problem of the curse of dimensionality [12, 13]; among them, several supervised techniques (i.e. Support Vector Machine, Random Forest, Bayesian classification) represent helpful tools for classification and biomarker discovery in clinical proteomics [14]. Here, we propose the application of multivariate data analysis, such as partial least squares discriminant analysis (PLS-DA), to perform biomarker selection on human protein microarrays. The PLS-DA [15, 16] model has the advantage of overcoming the so-called high dimensionality small sample problem [17] and it takes into account the noise in the system and multicollinearity [18]. As a result of these properties, in recent years, feature-ranking methods are being successfully applied in the field of gene expression analysis [19–22] but much less in the area of proteomics [23–30]. Here, using human protein microarray containing 1626 proteins selected throughout the human genome as described in our previous work [31] in combination with a bioinformatic approach based on the partial least squares discriminant analysis (PLS-DA), we investigate whether we can 1) perform a fast and more accurate selection for novel biomarkers and 2) confirm autoantibody responses of previously described biomarkers. For this purpose, a set of 263 serum samples were selected from 55 patients with autoimmune liver diseases, 95 patients with viral hepatitis, including 72 and 23 affected from hepatitis C virus and hepatitis B virus, respectively and 78 healthy donors (HD).

Two new AIH-specific autoantigens were identified, AHPA9419 and Chondroadherin precursor (UNQ9419 and CHAD, respectively) in addition to previously identified proteins. The two novel autoantigens were validated using a different sera cohort and receiver operating characteristic analysis showed a sensitivity ranging from of 65 to 87.5% and a specificity from 77.7% to 81.5%, respectively, for all control classes. These values are in line with the sensitivity of other selected autoantigens reported in literature [25, 32].

Herein, by combination of protein array and bioinformatics approach, two highly immunoreactive autoantigens, UNQ9419 and CHAD, with an AUC value greater than 0.70, were identified as specifically recognized by AIH patients. Interestingly, when the autoantigens were combined with the purpose to create a clinically valuable panel, the AUC showed a value of 0.915, while the sensitivity and specificity were 95.0% and 76.2%, respectively. These results indicate that the new autoantigens can be applied in clinical diagnostic and, together with the known biomarkers, can be used in the AIH diagnosis to improve sensitivity and accuracy.

## Materials and Methods

### Ethics statement

The use of biological material (such as serum) from healthy donors for research purposes and patients studies were approved by the Ethics Committee of IRCCS Ca’ Granda Policlinico Ospedale Maggiore in Milan, Italy and Sant’Orsola University Hospital, Bologna, Italy, respectively. Written informed consent regarding study participation was obtained from all involved adults or from the next of kin. Children were not be involved in the study. The study was approved by the Fondazione INGM Institutional Review Board (IRB).

### Serum Samples

Human sera samples were obtained from 263 individuals in two different hospitals: Sant'Orsola-Malpighi University Hospital, Bologna, Italy and IRCCS Ca' Granda Ospedale Maggiore Policlinico, Milan, Italy: 55 AIH, 72 HCV, 23 HBV and 113 healthy sobjects. The Table 1 reported clinical characterization of sera used for this study. AIH patients information from both discovery and validation studies were clinically diagnosed according the scoring system by the International Autoimmune Hepatitis Group and summarized in S1 Table (Supplementary Information section). Moreover, the level of IgG of the patients with AIH was higher than upper limit of normal with a median value of 1,1 (0.75–1.76), and a score of the 77%, and 17% (definite and probable respectively). All sample sera of patients with AIH were collected prior to treatment and were stored frozen in aliquots at -80°C. Each aliquot has been thawed no more than twice before use.

**Table 1:** Serum samples used in this study.

Phase	Group (Abbreviation)	Subtype (n)	n	Source^a^	Age: Mean± SD	Sex (n)
**Discovery**	Healthy Donors (HD)	__	*78*	1, 2	44 ±10	F(*20*) M(*58*)
	Autoimmune Hepatitis (AIH)	Type 1 (15) Type 2 (0)	*15*	2	50±21	F(*13*) M(*2*)
**Validation**	Healthy Donors (HD)	__	*35*	1, 2	48 ±8	F(*31*) M(4)
	Autoimmune Hepatitis (AIH)	Type 1 (33) Type 2 (7)	*40*	2	53±16	F(*34*) M(*6*)
	Viral hepatitis (VH)	^HCV (*72*) ^$^HBV (*23*)	*95*	2	52±12	F(*36*) M(*59*)

^a^Origin of samples: **(1)** IRCCS Ca’ Granda Ospedale Maggiore Policlinico,Milan, Italy, **(2)** Sant’Orsola University Hospital, Bologna.

^^^Patients affected from Hepatitis C Virus and

^$^Hepatitis B Virus

### Fabrication of the Protein Microarray and its probing with Human sera

The human protein microarray used in this study was composed of 1626 polyptides and was generated in INGM laboratory as previously described [31]. Briefly, after expression in *E*. *coli* as His-tagged fusions and purified from the bacterial insoluble fraction (0.5 mg/ml in 6M Urea), all human recombinant proteins were arrayed in a 384-well format and printed on nitrocellulose-coated slides (FAST slides, GE-Healthcare) in quadruplicate with the Microgrid II spotter (Biorobotics). A quality control of the spotting procedure was performed on 10% of randomly chosen slides. The percentage of proteins successfully spotted on the slides was assessed by hybridizing the microarrays with an α-His mAb, followed by an Alexa-647 conjugated α-Human IgG secondary antibody and estimating the number of spots with a mean fluorescence intensity (MFI) value significantly above background. The spotted microarrays were allowed to remain at room temperature for 1 h before storage at 4°C until use.

The probing procedure was identical for each microarray and conducted by TECAN Hybridization Station (HS 4800™ Pro; TECAN, Salzburg, Austria). The slides were blocked with BlockIt™ Microarray Blocking Buffer (ArrayIt Corporation), than diluted sera were incubated for 1 h at room temperature. Slides were then washed 3 times in PBST and probed for 1 h at room temperature with Alexa-647-conjugated anti-human IgG (Invitrogen). After washing steps at 25°C, the slides were finally dried at 30°C under nitrogen for 2 min.

### Scanning and image analysis

Protein microarray slides were scanned with a ScanArray Gx PLUS (PerkinElmer, Bridgeport Avenue Shelton, USA) and analyzed using ImaGene 8.0 software (Biodiscovery Inc, CA, USA). The fluorescence intensity of each spot was measured. To quantify signals we calculated the signal intensity for each protein spot, which was defined as the foreground mean intensity divided by its local background mean intensity. When the signal was <0 the protein spot was assigned the signal average of it for each group. We performed a within-array normalization for each slide on the basis of the signal distribution of all points of the human IgG curve using in-house developed software as previously described [33]. On the basis of these results, a normalized MFI value of 4.000 (value corresponding to the normalized MFI value of negative controls—BSA, HSA, Hu-GST- plus 2 standard deviations) was chosen as the lowest signal threshold for scoring a protein as positively recognized by human sera [31]. Moreover, for each protein, a Coefficient of Variation (CV%) was calculated on the four replicate spots for intra-assay reproducibility. If the CV% value was not within the expected range, the antigen was not considered for further analysis.

### Protein microarray data analysis

Each protein microarray is encoded by a vector of sorted features and each of them represents the normalized MFI of the pixels segmented as an autoantigen spot. Afterwards, the selected descriptors were organized in a quantitative feature matrix where rows and columns correspond to samples and autoantigens. To overcome the imbalance dataset issue the majority class was used through sampling several subsets independently the class itself, use these subsets to train classifiers separately and combine the trained classifiers into a final output (panel autoantigens). The female to male ratio of 3:1 was satisfied in the sampling selection of the majority class. 50, R, randomly partitioned datasets were generated to this aim while stability of protein profiles was assessed with Tanimoto index [34]. After computing the similarity index for each pair of feature preferences, the final stability was defined as the averaged over all pair wise similarity comparison between the fifty different protein sets (see Supporting Information).

### Robustness assessment of selection techniques

To perform the stability analysis two feature selection techniques, as well as Recursive Support Vector Machine (R-SVM) [35] and Partial Least Squares Discriminant Analysis (PLS-DA) [18, 36] were considered as representative of supervised feature selection methods. To assess the robustness of feature selection techniques, each feature selection algorithms was run on each generated R = 50 subsets, and the stability estimates were calculated within each datasets by 10 fold cross-validation results and the overall stability was averaged on all pair wise comparisons (see Supporting Information). The pseudo-code of the stability analysis is reported in S2 Table.

### PLS-DA, model validation and statistical analysis

**Fig 1:**
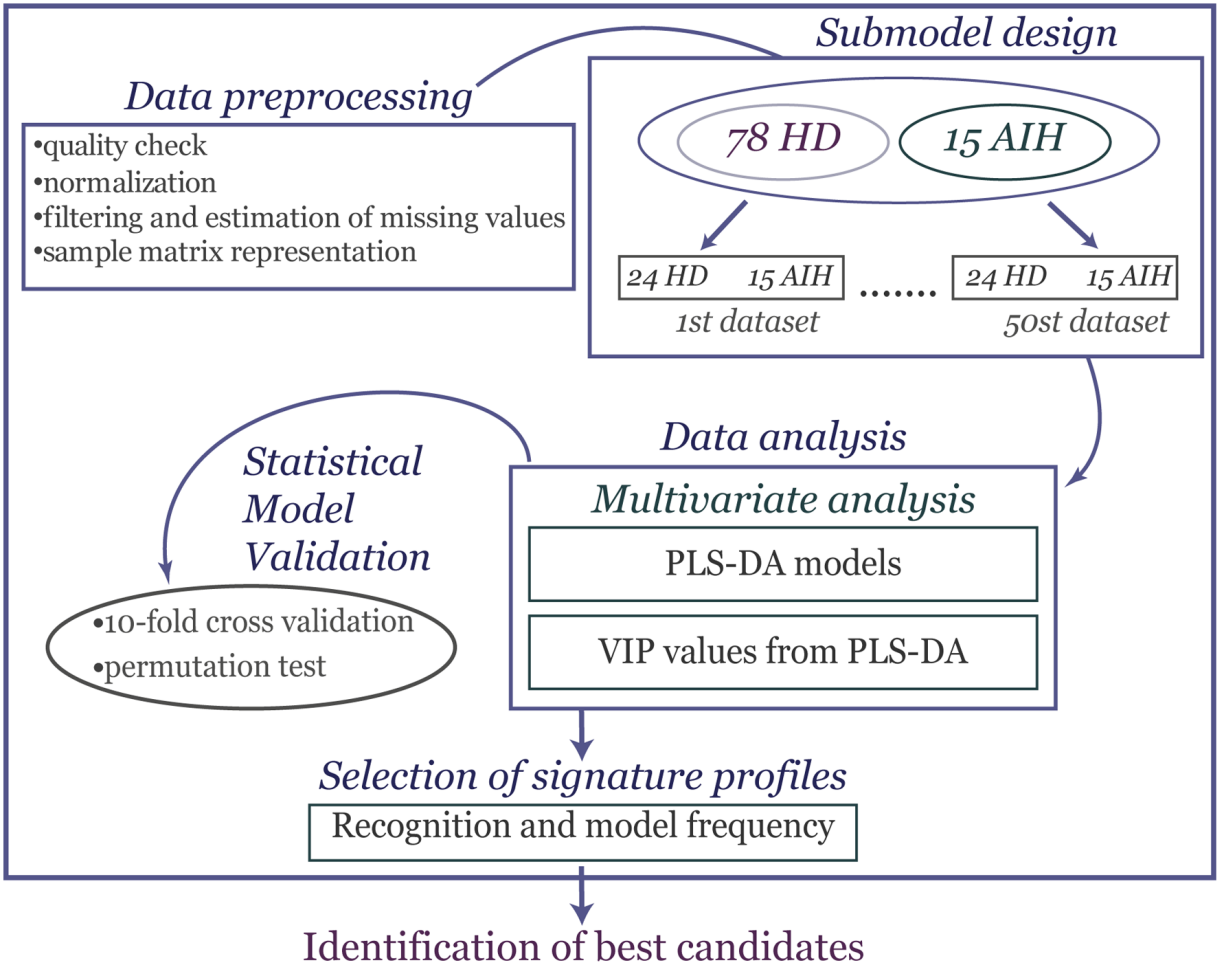
Workflow for signature selection profiles in protein microarrays. Protein microarray data were collected after scan image analysis and subsequently pre-processed and normalized. A submodel design included a 50 random data set generated to make balanced the comparison. Than PLS-DA was applied to visualize the separation between the two serum classes (AIH and HD) and to compute the discriminatory ability of each antigen to the separation based on the variable importance in the projection (VIP). To give a measure of the statistical significance of the diagnostic statistics a statistical model validation step was performed. In order to identify list of best candidates, a multi-criteria step based on recognition and relative frequency of autoantigens was applied.

PLS-DA was used for modelling the difference between AIH patients and HD controls [15, 16]. The model allows us to identify which descriptors explain most of the differences in the two groups by means of the variable influence in projection (VIP). The VIP is a weighted sum of squares of the PLS loading weights taking into account the amount of explained Y-variation in each dimension. The rule “greater than one” was used for detecting the descriptors with the greatest importance in the projection [18]. Validation of the PLS-DA model was checked using cross-validation (CV) and response permutation testing [14]. A CV procedure allowed us to randomly generate the necessary of training/test partitions from the original dataset. The differences between actual and predicted responses (calculated as reported in [36]) were calculated from all the parallel models to form PRESS (predictive residual sum of squares); this was a measure of goodness of prediction and generally it is re-expressed as Q^2^ (the cross-validated R^2^) which is 1-PRESS/SS where SS is the sum of squares of the response, corrected for the mean [18, 19]. One limitation of CV is that it provides over-optimistic results, so in order to give a measure of the statistical significance of the estimated power, Q^2^Y, (diagnostic statistic) and to test the model for over fitting due to chance correlation a permutation test was introduced. In this test only the labels of Y-block were randomly reordered while the X-block (antigen profiles) was intact. By repeating this procedure N times, a model is fitted to the new Y-data and new estimates of R^2^Y and Q^2^Y values were calculated [18, 36]. In this way, a reference distributions of R^2^Y and Q^2^Y are obtained, useful for appraising the statistical significance of such parameters; if “real” values are found outside such distributions this is a sign of high validity of model [18]. All computational analyses have been run within the R statistical language [37]. The plspm package (http://cran.r-project.org/web/packages/plspm/index.html) provides R functions for PLS-DA algorithm. Potential biomarkers were selected based on VIP> 1, the recognition frequency by less than 25% of the HD sera and more than 50% of AIH sera and the relative frequency of autoantigens over the all generated datasets of 100%. A summary of the proposed bioinformatic approach for proteomic biomarker discovery, described above, is summarized in Fig 1.

### Dissociation-enhanced Lanthanide Fluoroscence ImmunoAssay (DELFIA^®^) and data analysis

For the experimental verification of the antigens selected, DELFIA^®^ assay, a time-resolved fluorescence method, was used as described previously [31]. In brief, 20 μg/ml purified recombinant proteins (in 6M Urea) were coated in a 384-well format plates in duplicate with the Fredom-Evo Liquid Handling (Tecan). The plates were blocked with a blocking reagent (PerkinElmer) than diluted sera was incubated for 1 hour at 37°C. The plates were then washed 5 times with washing buffer (PerkinElmer) and probed for 30 min at room temperature in the dark with Europium-labeled α-human IgG serum (1:500 in diluting buffer, PerkinElmer). After washing, using Hydrospeed™ (TECAN), plates were left at room temperature for 10 min and finally read by an Infinite F200 PRO instrument (Tecan). Fluorescence intensity values higher than the mean of HD plus 1 SEM were considered as positive.

DELFIA^®^ results were analyzed using the two-tailed X^2^ test, the Student’s t test, the Fisher’s exact tests, or the analysis of variance test using either TIGR Multiexperiment Viewer and GraphPad software.

The Epicalc package was used to obtain the Receiver Operating Characteristic (ROC) curves of the models and the area under the curve (AUC) values.

STRING software (http://string-db.org) [38] was used to analyze the biological network of UNQ9419 and CHAD.

### Immunoblot Analysis

Five-hundred nanograms of AHPA9419 (UNQ9419) (secreted full length domain) was expressed by *E*. *coli* with the His10 tag, such as 500 ng of Chondroadherin (CHAD) (secreted domain) were resolved by 12% SDS-PAGE. The separated proteins were electrotransferred onto nitrocellulose membranes (Biorad) according to the manufacturer’s instructions. After blocking nonspecific binding sites with 5% nonfat milk powder diluted in PBS plus 0.1% Tween 20 (TPBS). The membranes were incubated with anti-His antibody (GE Healthcare) and secondary HRP-conjugated anti-mouse IgG (GE-Healthcare) were used to detect His-tagged recombinant proteins. The recombinant CHAD and UNQ9419 proteins were detected in the pooled serum from 6 cases of AIH and 6 control individuals (1:150), respectively O.N. at 4°C. After washes three times in TPBS, the secondary HRP-conjugated antibody (anti-mouse IgG; GE-Healthcare) was diluted 1:1000 in TPBS and incubated for 1 h at room temperature. Bound antibodies were detected by means of enhanced chemiluminescence (Super Signal West Pico Chemiluminescence Substrate) (Thermo Scientific) and detected with LAS-3000 (Fujifilm, Wayne, NJ).

## Results

### Quality check and pre-processing of protein microarray

**Fig 2:**
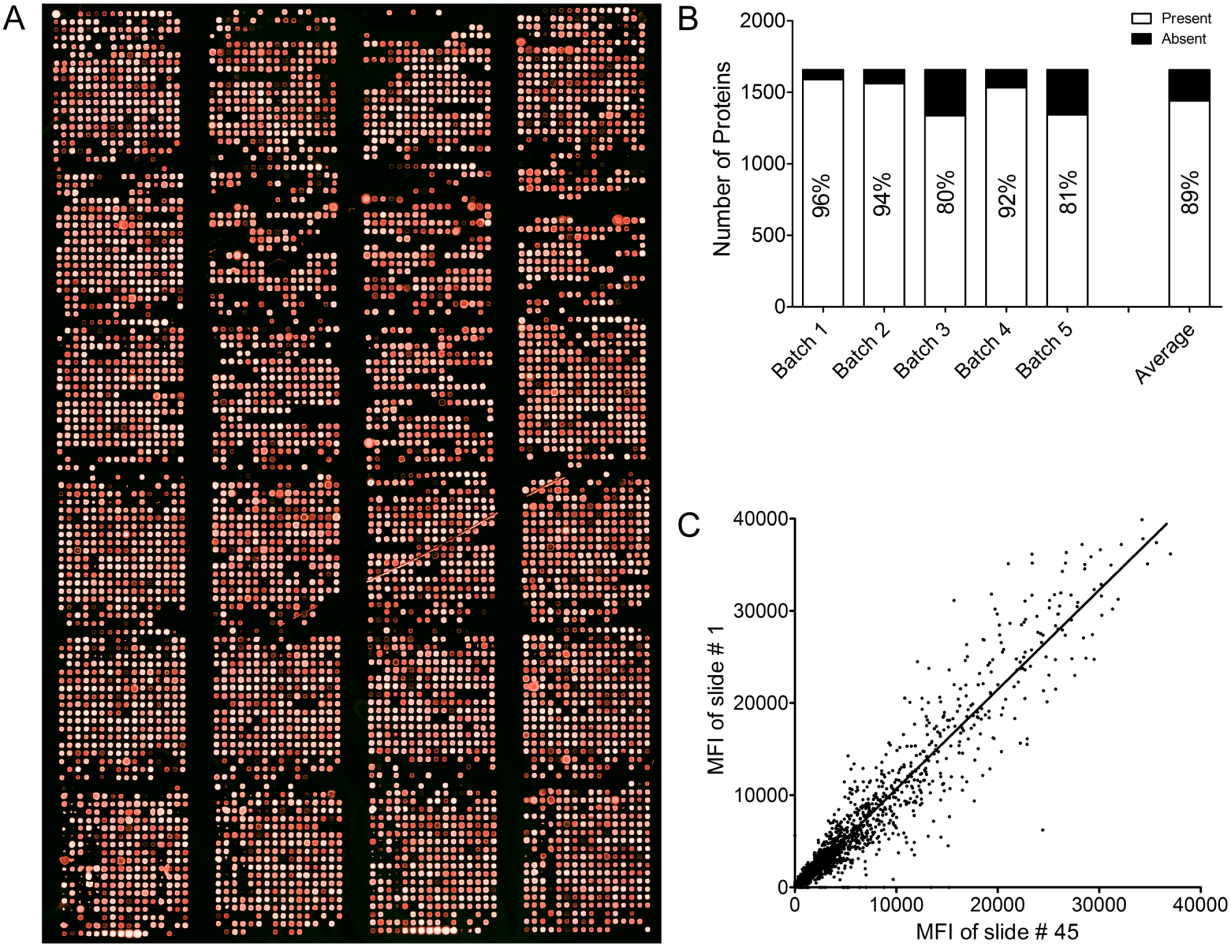
Human Protein Microarray: Quality check. (A) The protein microarray was probed with an anti-His mAb followed by a secondary anti-mouse antibody Alexa-647-labeled. Recombinant human proteins were purified and printed in quadruplicate on nitrocellulose slides. (B) Number of proteins detected with the anti-His mAb. About 90% of proteins were spotted with success on the slides (i.e about 90% of the proteins produced signals that were significantly above the background signal). Histograms: Proteins were considered “Present” when at least two out of the four replicates gave a signal above the background, otherwise they were considered “Absent”. (C) Correlation among spot intensities of two different slides (Slide 1 Vs Slide 45) of the same batch. The scatter plot indicates a positive correlation. The correlation coefficient is 0.9, indicating a high reproducibility of the signals derived from the proteins spotted.

To identify autoreactivity in patients with AIH, we used human protein microarrays carrying 1626 proteins, as previously reported [31]. The final protein microarray design consisted of 24 grids each of 304 spots, for a total of 7296 spots (Fig 2A).

The quality and quantity of the immobilized proteins on the microarray were determined by probing with an anti-His mAb and 89% of the proteins produced signals that were significantly above the background (Fig 2B). To identify potential AIH-associated autoantigens, we used ImaGene 8.0 to acquire the resultant signal intensities of all protein spots in each assay and an in-house developed software in order to select the positive spotted human proteins within each microarray prior normalization (see details in “Materials and Methods”). The results showed a high correlation among spot intensities of two different slides of the same batch indicating a high reproducibility of the signals derived from the spotted proteins (Fig 2C).

### Multivariate analysis and statistical model validation

In order to identify an AIH-associated signature profiles, we first evaluated the distribution of the Mean Fluorescence Intensity (MFI) generated from sera of AIH patients to HD subjects. AIH patients showed higher reactivity toward autoantigens than healthy donors (S1 Fig).

Then to further determine the overall feasibility of the bioinformatic strategy (see Fig 1), we carried out the analysis in two phases.

**Fig 3:**
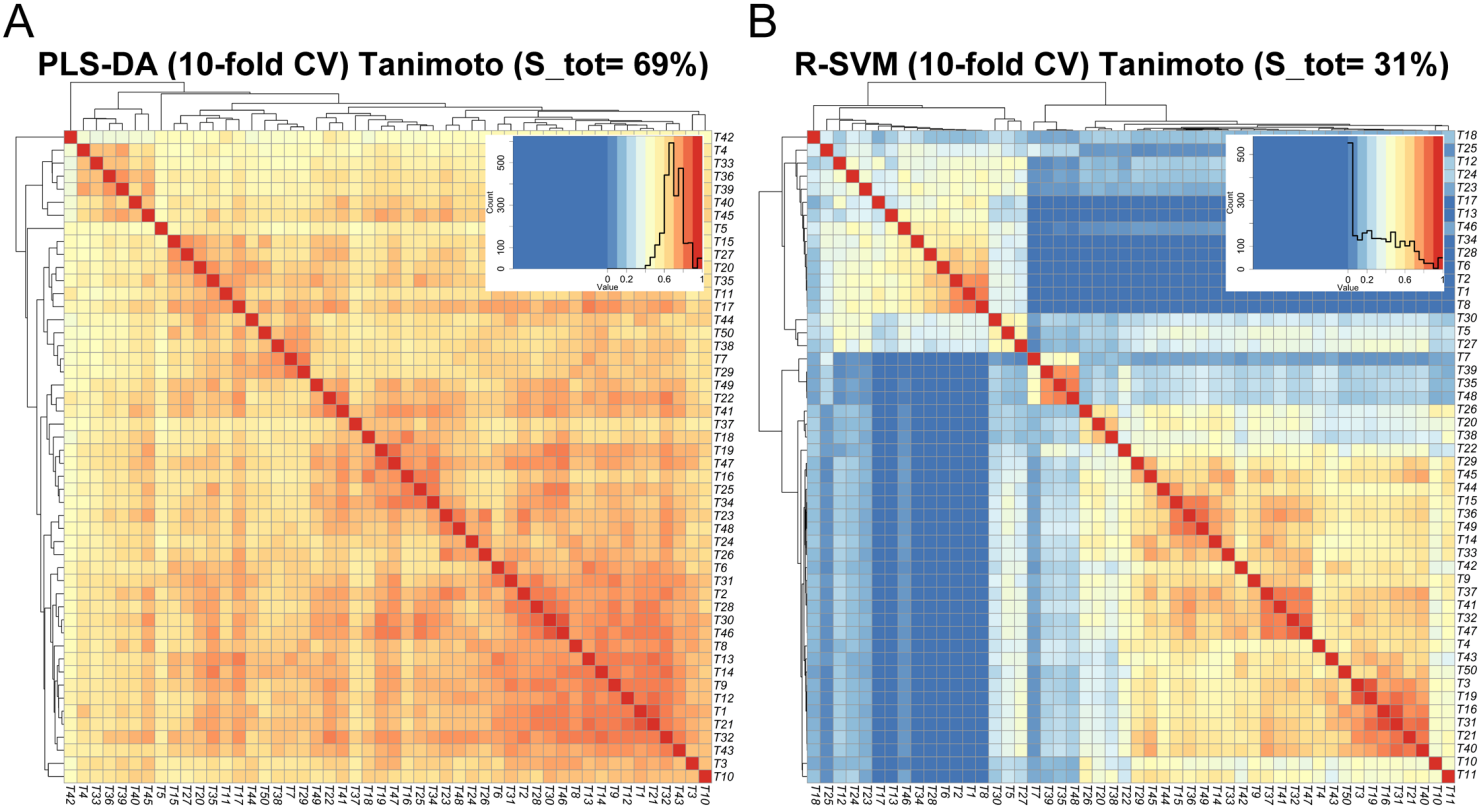
Evaluation of feature stability. Robustness of the R-SVM and PLS-DA rankers across the different 50 datasets is plotted as heat maps. Columns and rows represent the independent 50 subsets and each square indicates the Tanimoto index between two subsets. The colour code of the heat map ranges from blue to red where a blue colour reflects a low similarity index suggesting few proteins in common between the subsets while a red colour denotes an high similarity index. For each considered selection method a similarity heat map is obtained. (A), The average similarity over all pair wise comparison is 69% for PLS-DA; (B), and 31% for R-SVM; thus PLS-DA outperforms the R-SVM.

In the first phase, we conducted a data preprocessing and submodels design in order to overcome the imbalance problem of our samples and many (training) dataset were generated to get balanced ones fulfilling the female prevalence requirement (see Supporting Information). Moreover, since features extracted from different datasets might contain information of different aspects, we conducted a stability analysis comparing the ability of two rankers to select stable protein lists across different datasets, such as PLS-DA and R-SVM. The analysis revealed that PLS-DA outperforms R-SVM because it generated in average more similar protein lists. Specifically, the average similarity over all pair wise comparison is 69% for PLS-DA and 31% for R-SVM as showed in Fig 3.

**Fig 4:**
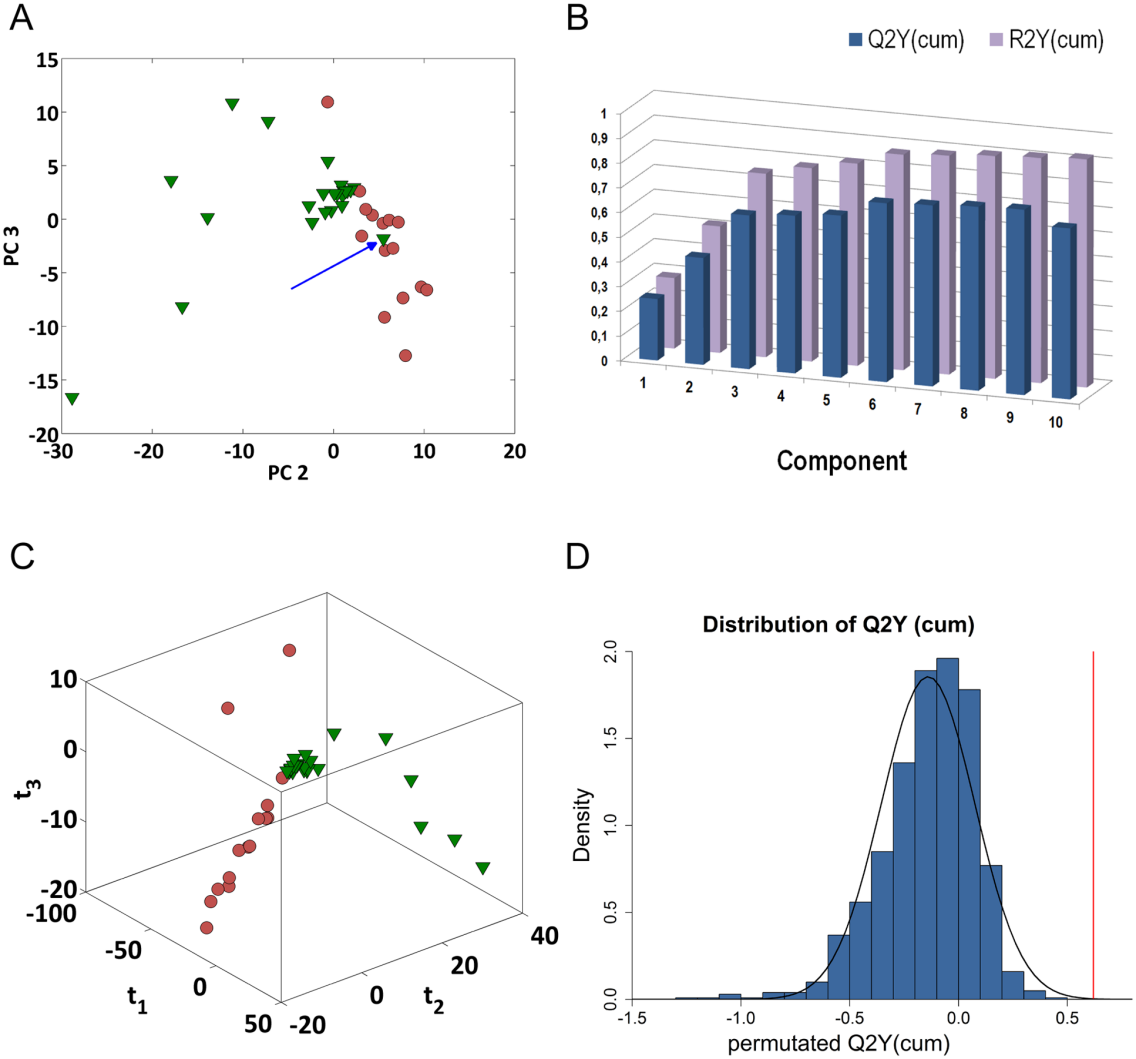
Overview of the PLS-DA analysis for the comparison between AIH and HD group. The normalized Mean Fluorescence Intensity (MFI) from microarray data was analyzed using PCA and PLS-DA models. (A) PCA shows that AIH (red circle) and HD (green triangle) have distinctive profiles with little overlap between the two groups of samples; the exception was the sample HD0088 (blue arrow) so this sample was omitted from the subsequently explorative analysis. (B) Plot of R^2^Y (explained variation) and Q^2^Y (predicted variation) shows how the considered parameters change as a function of increasing model complexity. Three components were calculated through cross-validation, R^2^Y and Q^2^Y were 74.28% and 62.18% and resulted significant in order to explain the relationship between the descriptor matrix and the class response. (C) PLS-DA 3D score plot reveals that each sample is found close to the samples belonging to the same subgroup. Samples are coloured according to the disease status (AIH- red circle, HD green triangle—the axes of the plot indicate PLS-DA component 1–3). (D) Density plot of the Q^2^Y values in the analysis of 1000 permutation tests, solid red line shows the real Q^2^Y value. Such reference distribution can be seen as sign of the degrees of overfit and overprediction of the model. The permutation test showed that the real PLS-DA model was not over-fitted and not over-predicted.

In the second phase, the PLS-DA was performed on all samples. However, in order to screen for outliers and to survey possible groupings [39] PCA was applied to the 50 balanced data sets. As shown in Fig 4A, the score plot of the PCA model, for one possible dataset (nAIH = 15, ñHD = 24), showed that AIH subjects were well separated from healthy controls with the exception of one outlying HD sample. Indeed, the exclusion of this sample provided in average more similar protein lists (S2 Fig); therefore, all variables related to this sample were removed from further analyses due to this atypical behaviour (see Supporting Information).

An overview of the analysis of one PLS-DA model is presented in Fig 4B and 4C; according to cross-validation three latent variables were sufficient to model the correlations within the dataset with R^2^Y and Q^2^Y of 74.28% and 62.19%, respectively (Fig 4B) and reported in S4 Table. The first three components depict a clear separation between the two groups according to their clinical conditions (Fig 4C). In particular, Fig 4D shows the Q^2^Y value for the original model in red and the reference distribution of Q^2^Y based on permuted data in blue for one of the considered dataset. It should be noted that the positive results of the model validation analysis give statistical relevance to the autoantigen changes suggesting that the non-expression related variations induced by experimental artefacts such as sample handling, are adequately compensated.

### Determination and evaluation of the biomarker candidates

**Fig 5:**
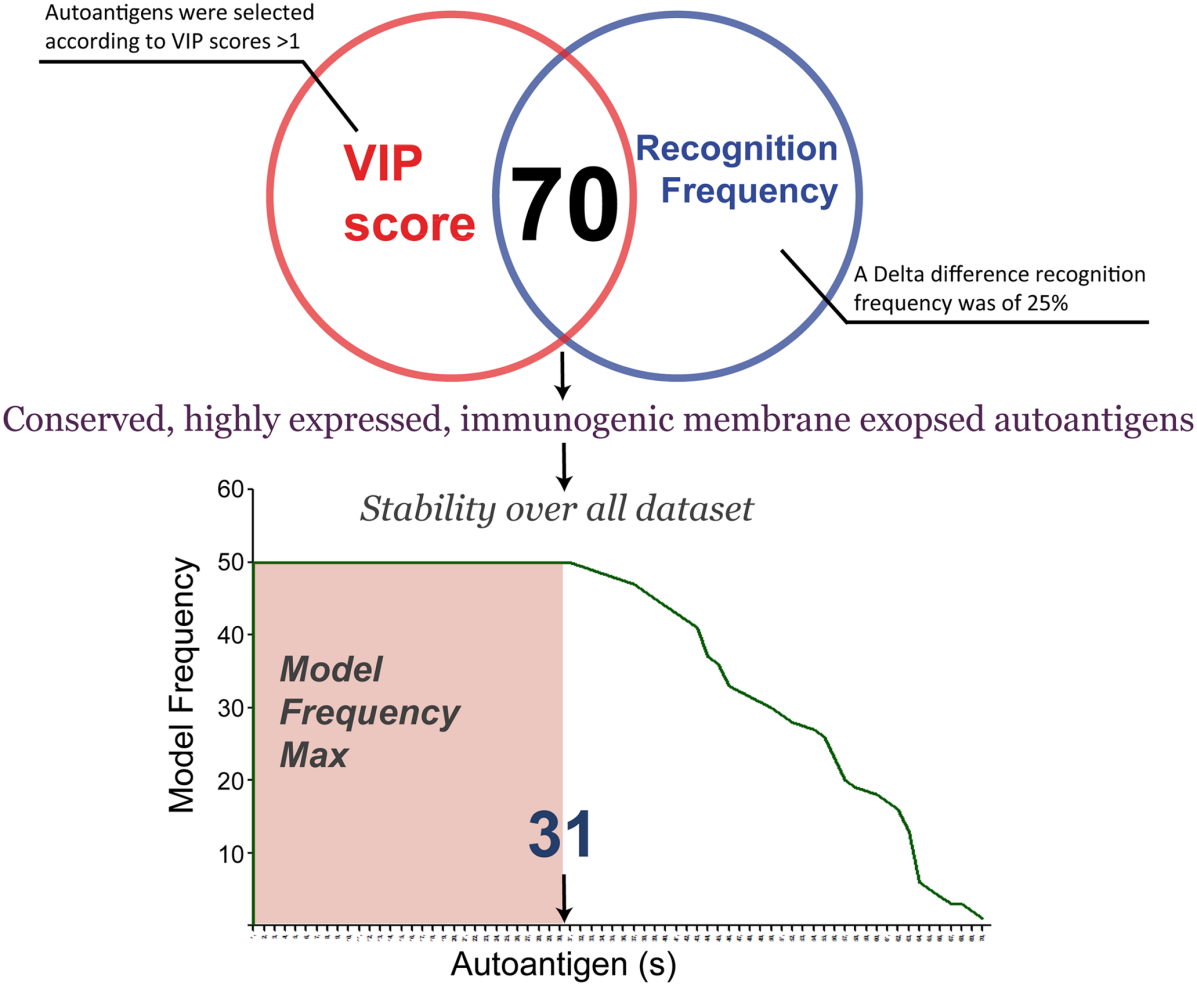
Selection strategy of serum autoantigens. The Venn diagram shows the autoantigen selection obtained according to (i) VIP scores >1.0 and (ii) a delta difference recognition frequency of 25%. 70 autoantigens overlap between the two filter criteria. Then, final selection of autoantigens was based on relative frequency of autoantigens from all generated datasets. There were 31 variables that were present in all datasets.

A multicriteria approach was used in order to define a targeted protein panel related to disease condition (Fig 5). First, the variable importance in the projection (VIP) values of the biomarker candidates was checked. Variables with VIP scores >1 were considered to have significant influence on the explanation of the separation. Second, self proteins were regarded as potential autoantigens if they were recognized by less than 25% of the HD sera and more than 50% of AIH sera. Seventy variable passed the above criteria. Third, autoantigens with a relative frequency (obtained from all generated datasets) less than 100% were excluded.

In this way, a list of 31 autoantigens was identified as discriminating between AIH subjects and healthy controls (Table 2). Interestingly, the strategy outlined gave promising and reliable results, indeed we confirmed that 87% (27 proteins) of these autoantigens were in common with those reported in our previous work [31] and 4 (13%) were selected as new autoantigens. These results confirmed that our approach is reliable. In order to confirm these 4 autoantigens, further validation in a different and larger population was used to assess the biological variation.

**Table 2:** Overview of autoantigens identified as potential signature profile of AIH. List of autoantigens involved in the discrimination between disease and healthy group. Using a VIP score>1 and a recognition frequency by less than 25% in HD and more than 50% in AIH a set of possible autoantigens was extracted. Features from all generated models were identified and compiled into a single list.

Description	Protein ID	VIP^	HD%	AIH%
*Interleukin 4 receptor*	IL4R	1.57	0	67
*Lysozyme g-like protein 1 Precursor*	LYG1	1.38	4	73
*Uncharacterized protein C19orf47*	C19orf47	2.04	4	80
*Solute carrier family 22 member 23*	SLC22A23	1.22	0	60
*Putative uncharacterized protein*	UNQ5830	1.94	0	80
*Putative uncharacterized protein DKFZp667F0711*	AL137145.1	1.91	1	87
*Hypothetical protein LOC648852*	LOC648852	1.32	0	60
*Putative uncharacterized protein*	LOC646100	1.68	0	73
*Uncharacterized protein C17orf99 Precursor*	C17orf99	1.46	0	73
*Uncharacterized protein C17orf99 Precursor*	C17orf99	1.6	0	67
*UPF0631 protein HSD24*	AC130289.1	1.23	0	60
*Transmembrane 95 Precursor*	TMEM95	1.12	0	60
*Cys-rich secr*. *prot*. *LCCL domain- containing 2*	CRISPLD2	1.21	1	60
*Ankyrin repeat domain- protein 43 Precursor*	ANKRD43	1.27	0	60
*RPE-spondin Precursor*	C8orf84	1.42	0	67
*Carboxypeptidase-like protein X2 Precursor*	CPXM2	1.83	0	80
*DnaJ homolog subfamily C member 30*	DNAJC30	1.44	4	73
*Chondroadherin-like Precursor*	CHADL	1.14	4	73
*Protein APCDD1-like Precursor*	APCDD1L	1.49	0	73
*Putative uncharacterized protein*	AC016586	1.45	0	60
*VGSA5840*	AC060225	1.3	0	60
*AHPA9419*	UNQ9419	1.36	0	53
*Calcium homeostasis modulator protein 3*	CALHM3	1.25	0	80
*T cell receptor beta variable 7*	A0A598	1.24	0	53
*Putative uncharacterized protein*	AC007245	1.33	0	67
*Thymic stromal cotransporter homolog*	SLC46A2	1.21	0	67
*Inhibin beta E chain Precursor*	INHBE	1.51	0	60
*WFDC10B Precursor*	WFDC10B	1.1	0	60
*R-spondin-3 Precursor*	RSPO3	1.08	0	53
*Membrane progestin receptor alpha*	PAQR7	1.5	0	80
*Chondroadherin Precursor*	CHAD	1.65	1	87

**HD:** healthy donors; **AIH:** Autoimmune hepatitis patients.

^^^Average over 50 dataset.

### Validation of novel AIH-associated autoantigens

**Fig 6:**
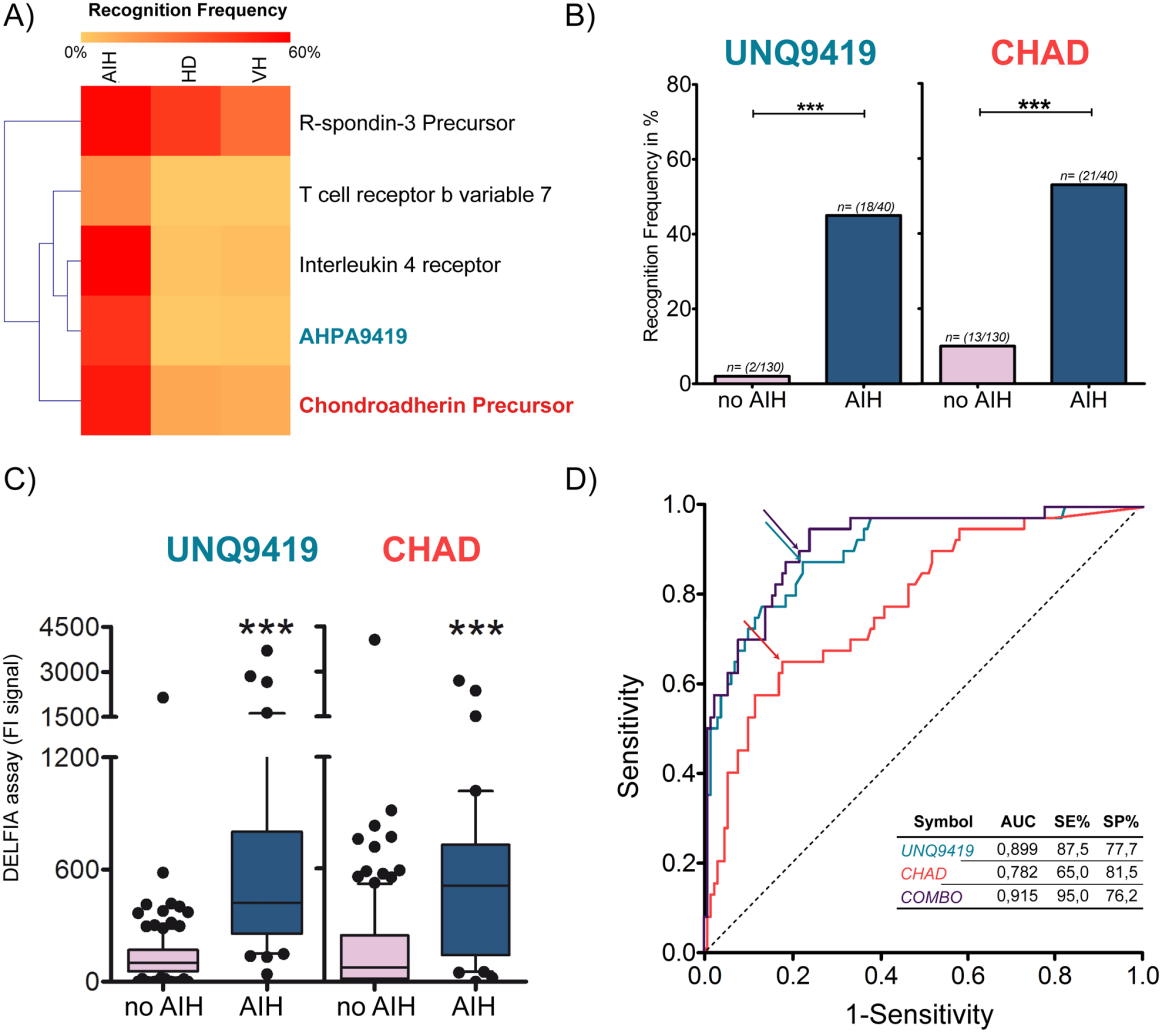
Evaluation of AIH-associated autoantigens. (A) The heatmap summarizes the recognition frequencies among different sample groups (AIH, HD and the VH) for the new four autoantigens out of the 31 candidates selected with the proposed bioinformatic strategy after DELFIA screening. Colour intensity denotes the degree of recognition frequency within the sample group. (B) Recognition frequency for two of the four autoantigens, that were statistical significant. (C) Signals distribution detected for each of the new two proteins are displayed. Statistical differences in recognition frequency (ChiSquare test) and in signal intensity (t test) are denoted as single (p<0,05), double (p<0,001) or triple stars (p <0,0001). (D), ROC curve of the biomarker candidates exhibited AUCs of 0.899 (SE = 87.5% and SP = 77.7%), 0.782 (SE = 65.0% and SP = 81.5%) for UNQ9419 and CHAD, respectively. The arrows denote best cut-off points. Combo curve represents the combination, UNQ9419+CHAD, which exhibited AUC of 0.915 (SE = 95.0% and SP = 76.2%).

To validate the new four additional autoantigens of the 31 candidates selected by PLS-DA, we determined their respective sensitivities and specificities for AIH with DELFIA^®^ screening using a larger and an independent sample set of sera, The sera used included 40 AIH and 35 healthy controls. Two out of four proteins were statistically significant, with a sensitivity more than 40%, (UNQ9419 45% and CHAD 53%), and showed a specificity of the 100% and 89% respectively, in terms of recognition frequency. Moreover, because the AIH is a liver-specific autoimmune disease, we determined their specificity using sera sample with liver disease from viral hepatitis. Ninety-five patients with chronic viral hepatitis (72 patients affected from Hepatitis C Virus, HCV and 23 from Hepatitis B Virus, HBV) were compared with the results of AIH sera as reported in Fig 6A and 6B. These results showed a high correlation also in terms of signal fluorescence intensities (see Fig 6C). Table 3 shows the sero-reactivity of the validation sample sera and reveals that the combination of the two statistical significant hits enhances sensitivity to 65%, but the specificity was unsatisfactory with a decreasing in the controls group. In addition, our best candidate IL4-R was included in the analysis as control and showed sensitivity and sensibility value higher than 60% in AIH patient sera, similar to the published results (Table 3).

**Table 3:** Overview of sensitivity and specificity in a validation step.

Description	Symbol	Combo	^a^SE %	^b^SP % HD	^c^SP % VH
*Interleukin 4 receptor domain*	*IL4R*		63	97	96
*AHPA9419*	*UNQ9419*	•	45	100	98
*Chondroadherin Precursor*	*CHAD*	•	53	89	91
*T cell receptor beta variable 7*	*TCR-B7*		18	100	99
*Combination*	*UNQ9419+ CHAD*		65	89	88

^**a**^ Sensitivity is defined as the true positive rate in %.

^**b**^ Specificity is defined as the true negative rate in Healthy Donor (HD)subject in %.

^**c**^ Specificity is defined as the true negative rate in Viral Hepatitis (VH)patients in %.

We next assessed the discrimination power of combination of the new autoantigens by variable ranking criterion AUC (area under the curve) or area under the “receiver operating characteristic” (ROC) curve which combines the sensitivity and specificity of a given marker for disease diagnosis which ranges from 0.5 (no discriminating power) to 1.0 (complete separation) [40]. Similar approach has been reported in a previous work, where Zingaretti *et al*. showed that the power of the combination of four antigens in discriminating AIH from healthy individuals was better than a single autoantigen [25]. Therefore, ROC analysis of two novel potential biomarkers was carried out to validate the newly selected autoantigens and examine their contribution to the prediction of AIH (Fig 6D). All samples without autoimmune hepatitis, including patients affected by HCV, HBV, and healthy individuals, were used as unique control class (here called no AIH).

The results of ROC analysis and the variables are ranked according to their AUC, either single and in combination. UNQ9419 had the highest AUC of 0.899. At the best cut-off point (Fig 6D) 87.5% sensitivity and 77.7% specificity were obtained. Autoantigens were then combined for the purpose of building a clinically valuable panel. The AUC showed a value of 0.915 while the sensitivity and specificity were 95.0% and 76.2%, respectively (Fig 6D). In all, these results indicated that the new autoantigens can be applied in clinical diagnostic and, together with the known biomarkers, can be used in the AIH diagnosis to improve the sensitivity and accuracy.

**Fig 7:**
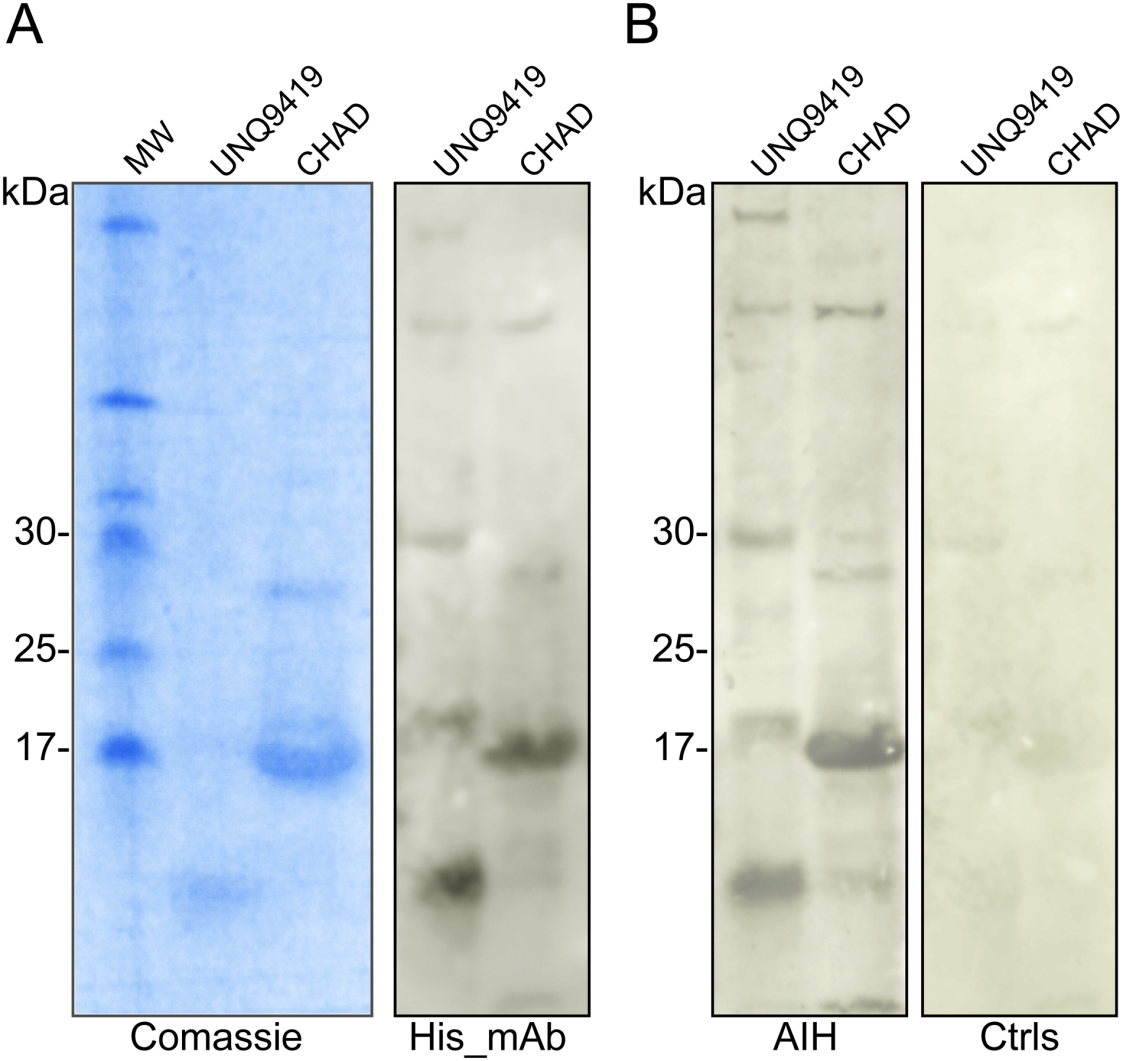
AIH sera recognize UNQ9419 and CHAD. (A), SDS-PAGE (left panel) and western blot against anti-His antibody analysis (right panel) of the purified UNQ9419 and CHAD recombinant proteins. (B), western blot analysis of the purified UNQ9419 and CHAD recombinant proteins against sera from AIH patients (left panel) and no AIH subjects (right panel), respectively.

Finally, the two novel autoantigens were also detected in the pooled serum from 6 AIH patients rather than no-AIH individuals, which indicated the capability of human sera to recognize the specific bands of UNQ9419 and CHAD. SDS-PAGE and western blot analysis, detected by anti-His antibody, showed that the bands of purified recombinant UNQ9419 and CHAD proteins appeared at 10.5 kDa and 18.2 kDa, respectively, which were similar to the expected molecular weight (Fig 7).

In order to analyze potential roles of UNQ9419 and CHAD in inflammatory disease, the intrinsic interactions with other proteins were analyzed using STRING software [38]. In the protein interaction maps obtained for CHAD twenty-one interacting proteins were predicted, including most interactions with several types of integrins, while for UNQ9419 a single interaction detected with low score (see S3 Fig).

## Discussion

We describe here the use of protein arrays in association with PLS-DA multivariate analysis in order to obtain a series of autoantigens recognized by autoantibodies in the serum of autoimmune hepatitis patients, and suggest that they could be used either alone and/or in combination as diagnostic markers. To achieve this goal we screened autoantibodies repertoires in 15 individuals with AIH and 78 healthy donors using a in house-developed protein array [31].

Specifically, we took into consideration the importance of the data analysis and potential hazards of interpretation. Indeed we wanted to improve the selection pipeline to highlight a complete and exhaustive list of discriminating features, overcoming the small sample size problem and applying a more rapid and accurate strategy. Proteomics dataset are characterized by few samples compared to the total number of variables and this undersampling can lead to problems such as features selection whose discriminatory power is purely due to chance or overfitting derived model which is specific for the selected dataset. The intrinsic characteristics of multivariate techniques coupled with a statistical validation strategies, such as cross-validation and permutation tests, make them ideal for the analysis of the proteomics datasets overcoming the many issues concerning the data analysis in this field. Differently from our previous work [31], we decided to focus on multivariate approaches simply because they get to capture feature redundancy and interaction neglected by univariate methods which evaluate each feature in isolation from all other and without any direct relation to the classification algorithm.

In order to provide a condensed signature profiles we also chose to apply a multi criteria based on recognition frequency and relative frequency of autoantigens over all datasets. Of note, this more representative panel, composed of 31 autoantigens, has a large number of discriminating features (about 87%) in common with the autoantigens seen in our previous work obtained by using a univariate data analysis [31]. The validity of these findings supports the feasibility of using supervised machine learning methods in combination with protein microarrays.

The reported results show that the outlined strategy is a valid support to proteomics studies. Firstly, the approach proved to be highly reliable and reproducible, providing a fast discrimination tool; additionally, protein microarray data are processed in automatic and unbiased procedure reducing errors; indeed it may highlight the presence of samples with divergent proteomic pattern then excluding them.

Six proteins in the newly selected list were already validated and showed high sensitivity (from 42% to 70% of positive AIH patients) and specificity (from 96% to 100% of negative HDs), as previously reported [31]. Among validated proteins, IL4 receptor autoantigen was studied for the neutralization role in autoimmune hepatitis, demonstrating that IL4R autoantibodies are functional in autoimmune hepatitis disease.

In this study we selected a list of proteins that included potential new biomarkers in autoimmune hepatitis and we validated four proteins that were not previously found to verify if they could be valid biomarkers. It was not surprising that there was not a completely overlap between the two selected list. Indeed, the two approaches are quite different: the traditional one [31] is based on the MFI of a single autoantigens related through the different microarrays whereas the described strategy, based on multivariate techniques, takes into account the feature interdependencies in the feature selection process. Moreover, it should be noted that these potential biomarkers differ from those discovered by other groups in previous studies [8, 9] because our protein array is designed to target either membrane-associated or secreted proteins, and great majority of which are poorly characterized and with unknown function [41].

Indeed, we were able to validated two new autoantigens for all control classes. ROC analysis shows that the putative biomarkers achieve a sensitivity and specificity ranging from 65% to 87.5% and from 77.7% to 81.5%, respectively. When UNQ9419 and CHAD were analyzed in combination the sensitivity and specificity were 95% and 76.2%, respectively.

One of the two new biomarker candidates is a protein with unknown function, annotated as AHPA9419 (UNQ9419), the other is a protein known as chondroadherin precursor (CHAD) (New UniProtKB accession number format in release 2014_06). CHAD is a short leucine rich-repeat protein (SLRP), a family of proteoglycans that have key roles as potent effectors in cellular signaling pathways [42]. Recent research studies have shown that SLRPs regulate biological functions in many tissues such as skin, tendon, kidney,liver, and heart [43, 44]. It has been also reported the role of decorin (a SLRP member protein) in extracellular matrix of liver fibrosis as inhibitor of TGF-β [45], the most powerful profibrotic cytokine, and studies suggest the presence of other SLPR members, such as CHAD, in attenuating TGF-β bioactivity [46–48]. In the web-network analysis we found that CHAD include mostly interactions with integrins, transmembrane receptors involved in the attachment of the cell to the extracellular matrix (ECM) and signal transduction from the ECM to the cell. It has been also shown that integrins mediated activation of TGF- β pathway in inflammation process [49]. Moreover, knowledge of the relationship between integrins and receptor tyrosine kinase has laid a foundation for new approaches to cancer therapy [50].

In this perspective further understanding of the possible pathological role of CHAD protein and presence of its autoantibodies in autoimmune hepatitis should pave the way of new therapies. Then, additional efforts will be useful for the identification and characterization of UNQ9419 protein.

## Conclusions

Autoimmune hepatitis is a complex disease characterized by the presence of circulating autoantibodies, hypergammaglobulinemia, necroinflammatory changes on hepatic histology and a dramatic response to immunosuppressive therapy [51]. A “biomarkers profile” containing a combination of benchmarks in clinical use and our best candidates [31] might help to better discriminate specific pathologies that share common features with autoimmune liver diseases and could be more informative for elucidating the pathology and clinical status [52]. The use of information from serum patterns of each patient will lead to the development of customized therapy.

## Supporting Information

S1 Fig Comparative profiling between analyzed classes. Representative Mean Fluorescence Intensity (MFI) distribution of healthy donor subjects (HD, top panel) compared with Autoimmune Hepatitis patients (AIH, bottom panel).
(PDF)

S2 Fig Stability comparison with and w/o HD0088. (A) We conducted a similarity analysis, based on PLS-DA with and (B) without HD0088 sample to strengthen the results obtained by PCA. Indeed, the exclusion of this sample provides in average more similar protein lists relative to the analysis with the cited sample, therefore, all variables related to this sample were removed from further analyses due to this ambiguous behaviour.
(PDF)

S3 FigProtein interaction network.Proteins involved in UNQ9419 and CHAD functions were analyzed using STRING software. Different line colours represent the types of evidence for the association predicted by different methods. -, shows a significant protein interaction from the reports of literatures; - shows a significant protein interaction groups gathered from databases; - shows protein interaction groups extracted from scientific literatures. (A) This network received a low score. Predicted functional partner was only one protein: KIAA1524, an oncoprotein that inhibits PP2A and stabilizes MYC in human malignancies. Promotes anchorage-independent cell growth and tumour formation. (B) This network received a score of more than 0,8. The predicted function includes integrins, transmembrane receptors that are the bridges for cell-cell and cell-extracellular matrix (ECM) interactions. 10/21 interacting proteins for CHAD are depicted as: ITGA2, integrin, alpha 2; ITGA4, integrin, alpha 4; ITGA5, integrin, alpha 5; ITGA6, integrin, alpha 6; ITGB1, integrin, beta 1; ITGB5, integrin, beta 5; ITGB6, integrin, beta 6; ITGA8, integrin, alpha 8; ITGA10, integrin, alpha 10; ITGA11, integrin, alpha 11.(PDF)

S1 Table Antibody response and clinical characteristics of AIH patients. (PDF)

S2 Table Pseudo-code of the robustness of feature selection algorithms. (PDF)

S3 Table Model performance of PLS-DA models for the AIH vs HD comparison. Summary statistics of the PLS-DA models for the fifty dataset. A cross-validation strategy was employed to give an estimate of the significance of a latent variables; an appropriate number of components is given where model have an optimal balance between fit (R^2^Y, explained variation) and predictive ability (Q^2^Y, predicted variation). Most models are well modelled after three PLS components, but there are some exceptions.
(PDF)

S4 Table Summary statistics of one PLS-DA models for the AIH vs HD comparison. Overall R^2^Y and Q^2^Y statistics change as a function of increasing model complexity (for one of the fifty generated submodels). Here the cross-validation procedure suggests that three components are appropriate to explore the correlations within dataset. The three components explain 74.28% (R^2^Y = 0.74) and predict 62.19% (Q^2^Y = 0.62) of the variation in the response variable.
(PDF)

S1 Text Protein Microarray data analysis. (PDF)
