# Supplementary figures and images for: Two of Them Do It Better: Novel Serum Biomarkers Improve Autoimmune Hepatitis Diagnosis
Source: PLoS One. 2015 Sep 16;10(9):e0137927. doi: 10.1371/journal.pone.0137927 (PMC4573979; doi:10.1371/journal.pone.0137927)

**S1 Fig.**

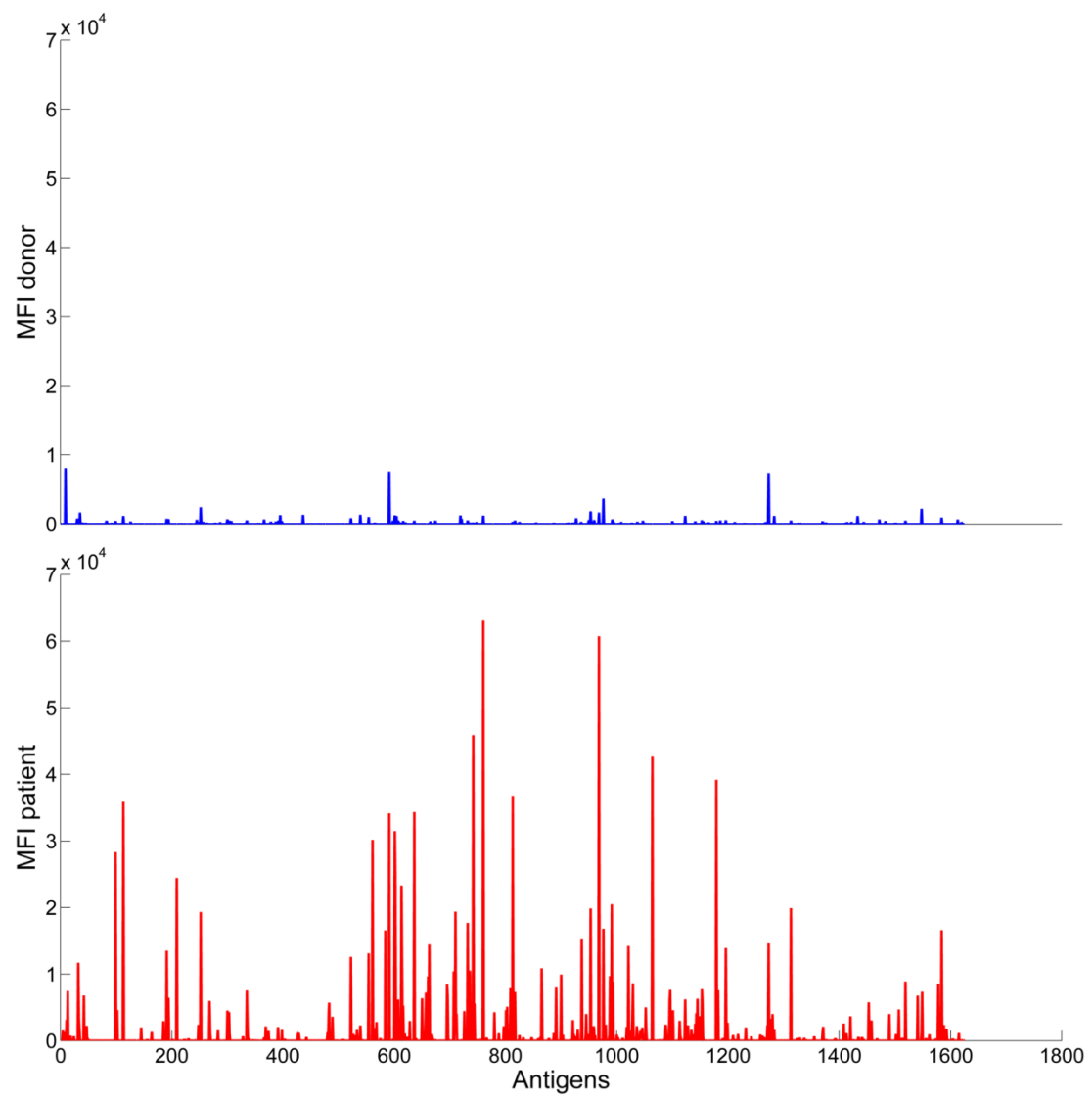

Supplement: S1 Fig — Representative Mean Fluorescence Intensity (MFI) distribution of healthy donor subjects (HD, top panel) compared with Autoimmune Hepatitis patients (AIH, bottom panel). (PDF) [file pone.0137927.s001.pdf]

S2 Fig.

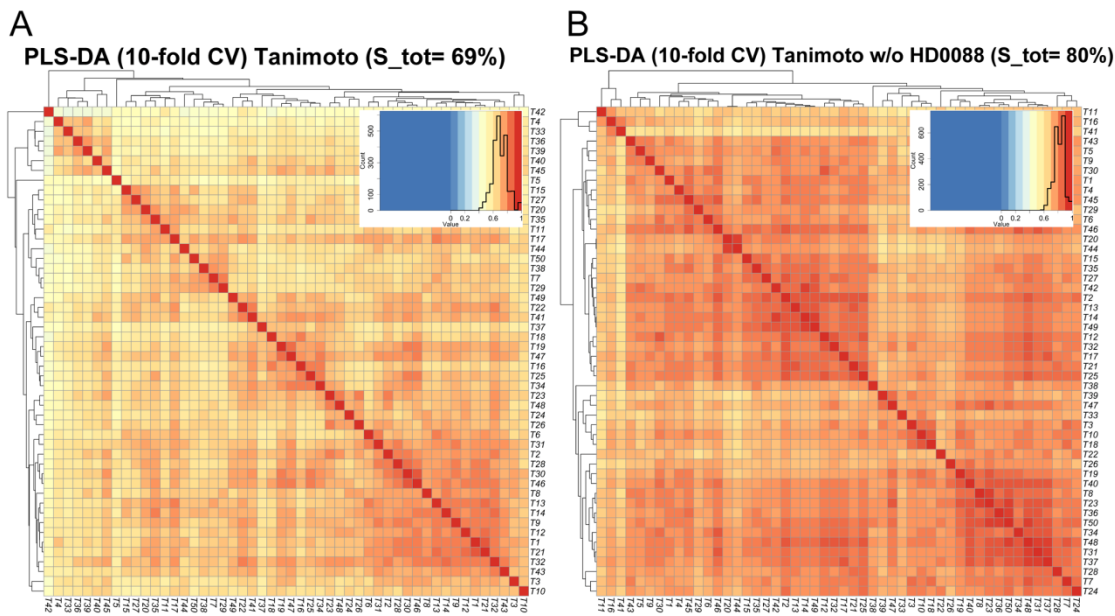

Supplement: S2 Fig — (A) We conducted a similarity analysis, based on PLS-DA with and (B) without HD0088 sample to strengthen the results obtained by PCA. Indeed, the exclusion of this sample provides in average more similar protein lists relative to the analysis with the cited sample, therefore, all variables related to this sample were removed from further analyses due to this ambiguous behaviour. (PDF) [file pone.0137927.s002.pdf]

**S3 Fig.**

**A**

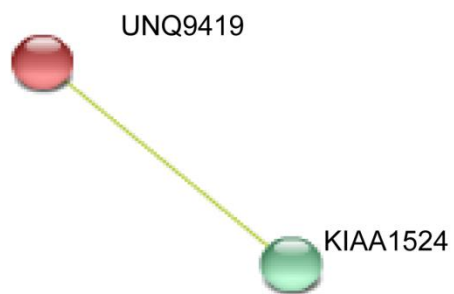

**B**

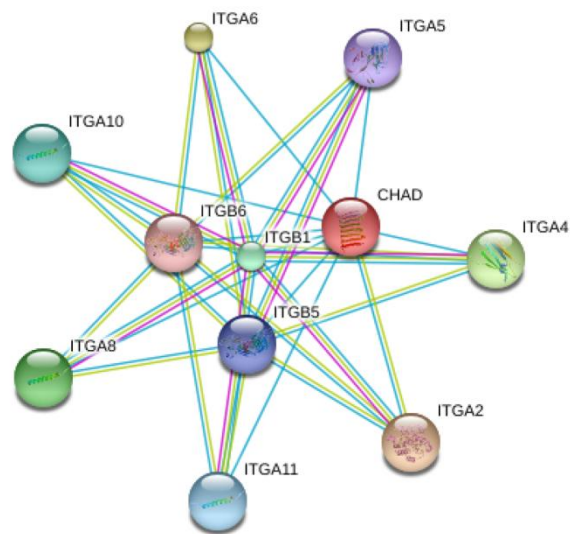

Supplement: S3 Fig — Proteins involved in UNQ9419 and CHAD functions were analyzed using STRING software. Different line colours represent the types of evidence for the association predicted by different methods. -, shows a significant protein interaction from the reports of literatures; - shows a significant protein interaction groups gathered from databases; - shows protein interaction groups extracted from scientific literatures. (A) This network received a low score. Predicted functional partner was only one protein: KIAA1524, an oncoprotein that inhibits PP2A and stabilizes MYC in human malignancies. Promotes anchorage-independent cell growth and tumour formation. (B) This network received a score of more than 0,8. The predicted function includes integrins, transmembrane receptors that are the bridges for cell-cell and cell-extracellular matrix (ECM) interactions. 10/21 interacting proteins for CHAD are depicted as: ITGA2, integrin, alpha 2; ITGA4, integrin, alpha 4; ITGA5, integrin, alpha 5; ITGA6, integrin, alpha 6; ITGB1, integrin, beta 1; ITGB5, integrin, beta 5; ITGB6, integrin, beta 6; ITGA8, integrin, alpha 8; ITGA10, integrin, alpha 10; ITGA11, integrin, alpha 11. (PDF) [file pone.0137927.s003.pdf]
