# Supplementary material for: Two of Them Do It Better: Novel Serum Biomarkers Improve Autoimmune Hepatitis Diagnosis
Source: PLoS One. 2015 Sep 16;10(9):e0137927. doi: 10.1371/journal.pone.0137927 (PMC4573979; doi:10.1371/journal.pone.0137927)
Supplement: S1 Table — (PDF) [file pone.0137927.s004.pdf]

S1 Table.

Sample ID	Type	ANA	SMA	SLA	LKM1	LC1	anti-UNQ9419	anti-CHAD
PT001*	1	+	+	-	-	-	-	+
PT002*	1	+	+	-	-	-	+	+
PT003*	1	+	+	+	-	-	+	+
PT004*	1	+	+	na	-	-	+	+
PT005*	1	+	+	na	-	-	-	-
PT006*	1	+	+	-	-	-	+	+
PT007*	1	+	+	na	-	-	-	+
PT008*	1	+	+	na	-	-	+	+
PT009*	1	+	+	na	-	-	-	+
PT010*	1	+	+	na	-	-	-	-
PT011*	1	+	+	+	+	-	-	+
PT012*	1	+	+	na	-	-	-	+
PT013*	1	+	+	na	-	-	+	+
PT014*	1	+	+	-	-	-	+	+
PT015*	1	+	+	-	-	-	+	+
PT016	1	+	-	+	-	-	-	-
PT017	2	+	-	-	+	+	-	-
PT018	2	-	-	-	+	-	-	-
PT019	1	+	-	-	-	-	-	-
PT020	1	-	+	-	-	-	+	+
PT021	1	-	+	+	-	-	-	+
PT022	1	+	-	-	-	-	+	-
PT023	1	+	-	-	-	na	-	+
PT024	1	+	+	-	-	na	-	-
PT025	1	+	-	-	-	na	+	+
PT026	1	-	na	-	-	na	+	+
PT027	1	+	-	na	-	-	-	-
PT028	1	+	+	na	-	-	-	+
PT029	1	-	+	+	-	-	-	+
PT030	2	-	-	na	+	-	-	+
PT031	1	+	+	na	-	-	+	+
PT032	1	+	+	na	-	-	+	+
PT033	1	+	-	na	-	-	+	+
PT034	1	+	-	na	-	-	+	+
PT035	1	+	+	na	-	-	-	+
PT036	2	-	-	-	+	-	+	+
PT037	1	+	+	-	-	-	+	-
PT038	1&2	+	-	na	+	-	+	+
PT039	1&2	-	+	na	+	-	+	-
PT040	1	+	-	na	-	-	-	-
PT041	1	-	+	-	-	-	-	-
PT042	2	-	-	-	+	+	+	-
PT043	1	+	+	na	-	-	+	-
PT044	1	-	+	na	-	-	-	-
PT045	1	-	+	+	-	-	-	-
PT046	2	-	-	-	+	-	-	-
PT047	1	+	-	+	-	-	-	-
PT048	1	-	-	+	-	-	+	+
PT049	1	+	-	-	-	-	+	+
PT050	1	-	+	na	-	-	+	+
PT051	1	-	+	na	-	-	-	+
PT052	2	-	-	-	+	-	-	-
PT053	2	-	-	-	+	-	+	+
PT054	1	+	+	na	-	-	-	-
PT055	2	-	-	-	+	-	+	+

ANA: anti-nuclear antibodies; SMA: anti-smooth-muscle antibodies; LKM1: liver/kidney microsomal antibody type 1; SLA: anti-soluble liver antigen; LC1: Anti-liver cytosolic antigen type 1 antibody.
*Patient Sera used in the discovery phase.
