# Supplementary material for: Two of Them Do It Better: Novel Serum Biomarkers Improve Autoimmune Hepatitis Diagnosis
Source: PLoS One. 2015 Sep 16;10(9):e0137927. doi: 10.1371/journal.pone.0137927 (PMC4573979; doi:10.1371/journal.pone.0137927)
Supplement: S2 Table — (PDF) [file pone.0137927.s005.pdf]

S2 Table.

Similarity evaluation
INPUT: a dataset, D, with A features (autoantigens) and N instances ($n_{AIH}=15$ and $n_{HD}=78$ measurements).<br>R is the number of reduced subsets
For each run *i*, *i=1,2,.........R*<br><br>(1) randomly partition data into reduced subsets D_i including all positive instances ($n_{AIH}=15$) and a set of negative instances ($\tilde{n}_{HD}=24$)<br>(2) perform feature selection<br>*(i)* train y classifiers (*y=2*) with a *10 fold cross-validation* on the reduced subset<br>*(ii)* obtain a ranked list L_{iy}<br>End<br><br>Finally, calculate all pairwise similarity comparison (R(R-1))/2 possible comparisons) and averaged them to obtain an overall evaluation of the degree of similarity, *S*, between the autoantigen lists
OUTPUT: similarity index S_{tot} for each considered classifier
