# Supplementary material for: Two of Them Do It Better: Novel Serum Biomarkers Improve Autoimmune Hepatitis Diagnosis
Source: PLoS One. 2015 Sep 16;10(9):e0137927. doi: 10.1371/journal.pone.0137927 (PMC4573979; doi:10.1371/journal.pone.0137927)
Supplement: S3 Table — Summary statistics of the PLS-DA models for the fifty dataset. A cross-validation strategy was employed to give an estimate of the significance of a latent variables; an appropriate number of components is given where model have an optimal balance between fit (R2Y, explained variation) and predictive ability (Q2Y, predicted variation). Most models are well modelled after three PLS components, but there are some exceptions. (PDF) [file pone.0137927.s006.pdf]

S3 Table.

N. dataset	$R^2X(cum)(\%)$	$R^2Y(cum)(\%)$	$Q^2Y(cum)(\%)$	LV
1	71.31	73.39	55.90	3
2	70.24	74.55	53.15	3
3	69.27	72.66	61.85	3
4	69.35	72.49	59.91	3
5	73.60	73.81	59.29	3
6	72.40	74.25	55.29	3
7	69.78	73.81	62.20	3
8	71.51	73.03	62.48	3
9	74.55	73.92	61.40	3
10	70.87	72.92	50.83	3
11	64.16	62.87	55.53	2
12	72.08	72.98	63.40	3
13	70.64	74.26	56.85	3
14	70.89	74.34	58.23	3
15	71.45	73.76	61.40	3
16	62.76	61.72	53.31	2
17	70.71	72.53	55.30	3
18	68.62	74.28	62.19	3
19	69.41	74.14	58.70	3
20	70.90	74.46	58.23	3
21	69.55	74.02	54.89	3
22	65.23	62.23	55.92	2
23	70.07	71.02	62.23	3
24	69.63	73.95	56.86	3
25	69.45	74.86	64.47	3
26	68.52	71.75	59.43	3
27	69.92	74.64	59.25	3
28	70.47	73.33	58.48	3
29	69.07	75.83	60.18	3
30	70.06	73.08	56.47	3
31	71.67	72.34	60.09	3
32	71.01	72.77	59.69	3
33	68.50	71.40	56.80	3
34	69.60	74.82	64.57	3
35	72.02	74.16	63.22	3
36	70.36	71.52	61.09	3
37	68.76	75.54	61.72	3
38	70.86	73.45	58.62	3
39	67.62	72.89	61.23	3
40	70.12	71.89	55.37	3
41	63.50	63.84	61.26	2
42	73.99	74.04	55.87	3
43	72.94	73.52	59.48	3
44	69.97	75.14	64.40	3
45	68.83	72.68	59.67	3
46	71.33	74.68	59.77	3
47	71.16	72.19	59.05	3
48	68.88	74.49	58.81	3
49	70.57	74.31	47.62	3
50	71.29	70.71	58.49	3

R^2X, R^2Y: fraction of the variance of descriptor matrix (X) and class response (Y) explained by each latent variables (LV) in %, Q^2Y: fraction of the variance predicted (cross-validated) in %; $R^2X(cum)$, $R^2Y(cum)$: cumulative explained variation and $Q^2Y(cum)$ predicted variation in %.
