# Supplementary material for: Two of Them Do It Better: Novel Serum Biomarkers Improve Autoimmune Hepatitis Diagnosis
Source: PLoS One. 2015 Sep 16;10(9):e0137927. doi: 10.1371/journal.pone.0137927 (PMC4573979; doi:10.1371/journal.pone.0137927)
Supplement: S4 Table — Overall R2Y and Q2Y statistics change as a function of increasing model complexity (for one of the fifty generated submodels). Here the cross-validation procedure suggests that three components are appropriate to explore the correlations within dataset. The three components explain 74.28% (R2Y = 0.74) and predict 62.19% (Q2Y = 0.62) of the variation in the response variable. (PDF) [file pone.0137927.s007.pdf]

S4 Table.

LV	$R^2Y(cum)(\%)$	$Q^2Y(cum)(\%)$
t1	28.54	25.25
t2	51.20	44.25
t3	74.28	62.19
t4	78.24	63.68
t5	81.83	65.62
t6	87.21	71.55
t7	88.52	72.64
t8	90.09	74.16
t9	91.09	74.88
t10	92.29	69.09

$R^2Y(cum)$: cumulative explained variation of the class response (Y) explained by each latent variables (LV) in %, $Q^2Y(cum)$: cumulative predicted variation (cross-validated) in %.
