# Supplementary material for: Two of Them Do It Better: Novel Serum Biomarkers Improve Autoimmune Hepatitis Diagnosis
Source: PLoS One. 2015 Sep 16;10(9):e0137927. doi: 10.1371/journal.pone.0137927 (PMC4573979; doi:10.1371/journal.pone.0137927)
Supplement: S1 Text — (PDF) [file pone.0137927.s008.pdf]

Protein Microarray data analysis

Stability analysis: The discovery dataset includes 93 serum samples. These serum samples belong to two different groups: n=78 samples categorized as healthy subjects and n=15 categorized as AIH patients, suggesting the imbalance nature of the analyzed datasets. In imbalance datasets the class having more instances is defined major class while the one having relatively less number of instances is defined minor class [1]. Imbalance has relevant consequences on the learning process, usually producing classifiers that show poor predictive accuracy for the minority class and that tend to classify new samples in the majority class and ignore the minor class [1]. The bias is even more relevant for high-dimensional data, where the number of variables greatly exceeds the number of samples. In the last few years various techniques have been proposed to solve the problem of imbalance distribution of sample [2]; these approaches are mainly dividing into three categories such as sampling, algorithms and feature selection. The problem can be attenuated by undersampling or oversampling, which produce class-balanced data. This method leads to loss of valuable information owing to reduce sampling of the majority class in the training of classifier. To overcome this issue we proposed to make better use of the majority class through sampling several subsets independently from the majority class, to use these subsets to train classifiers separately and combine the trained classifiers into a final output (panel autoantigens). This approach outperforms better than simple undersampling, since multiple subsets contain more information than single one.

Given these different versions of the original data, it was necessary to evaluate the effect of instance perturbation on the feature selection results [3] because the results tend to be unstable. Indeed, it is now well documented that it is possible to find different feature sets which however produce similar prediction patterns [4]. This characteristic translates into a lack of stability and robustness of the protein expression signatures, making the selection of sets with relevant features for a classification task a critical issue and, at the same time, rendering their biological interpretation challenging.

To address these needs we followed a strategy proposed by Kalousis *et al.* where has been defined the stability of a feature selection algorithm as the robustness of the "feature preferences" it produces to training set perturbations [5]. Measuring stability requires a similarity measure for feature preferences.

We used the Tanimoto index to evaluate the degree of similarity/dissimilarity among the protein lists [6], which measures the amount of overlap between two sets of arbitrary cardinality. It ranges between 0 meaning no overlap between the two sets and 1 meaning two sets are identical. So, the similarity of each pair of features sets (with R subsets R(R-1)/2 pairs are possible) is computed using the Tanimoto index. More similar all subsets are, higher the similarity measure will be. In this work, two feature selection techniques were considered to perform the stability analysis. Recursive Support Vector Machine (R-SVM) [7] and Partial Least squares Discriminant Analysis (PLS-DA) were chosen as representative of supervised feature selection methods. R-SVM is a modified support vector machine algorithm which performs feature selection while builds the classifier in a multiple-step recursive manner following a given descendant ladder; the details of the method have been described by Zhang *et al.*; 2006.

Model-based multivariate analysis: For each of the fifty generated dataset a PCA model was fitted and when this outlier was present, it has been removed from the successive analysis. A two-class PLS-DA modeling has, therefore, been based, therefore, on this neatened dataset. A dummy matrix of two Y-variables [8] expressing diagnosis of the sera samples was created. Fifty PLS-DA models were calculated with different subset of HD samples and the values of the parameters (R^2X, R^2Y and Q^2Y) are presented in Supplementary Table S3. These parameters were positive indicating the existence of a robust discriminative pattern between AIH and HD samples. In PLS-DA, the R^2Y and Q^2Y parameters were used for the evaluation of the models, indicating the fitness and prediction ability. The explanatory ability of the model increases with the number of the components whereas the predictive ability of the model begin to decrease after a certain number of components.

Statistical model validation: One limitation of cross-validation is that it assess only the predictive power, but it is unknown which Q2 value corresponds to a valid model and if there is a statistically relevant difference between the groups. Therefore, a permutation testing was employed to give a measure of the statistical significance of the diagnostic statistics [9-11]. The approach produces a distribution of Q^2 values suitable for testing the null hypothesis for a model's

Q^2; a reliable model should yield a significantly larger Q^2 value compared to Q^2 generated from random models using the same dataset [12]. For the 50 datasets, we randomly permuted the labels of the response matrix many times (1000) and computed the new classification models; in this way a null reference distribution, that is expected not significant, was obtained for each performance parameter (R^2Y, Q^2Y). The results of response permutation testing offered a favorable picture: for the 1000 datasets in which the labels were randomly permuted the distribution of R^2Y and Q^2Y was always substantially lower than the corresponding real values. Hence it can be concluded that it is impossible to obtain models with the same predictive values by chance and neither of the models over fit the data.

Supporting Information References

1. Chawala NV, Japkowicz N, Kolcz A (2004) Editorial: Special issue on learning from imbalanced data sets. ACM SIGKDD explorations 6: 1-6.
2. Seiffert C, Khoshgoftaar TM, Hulse JV, Napolitano A (2008) A comparative study of data sampling and cost sensitive learning IEEE International Conference on Data Mining Workshops 15-19 Dec: 46-52.
3. He Z, Yu W (2010) Stable feature selection for biomarker discovery. Comput Biol Chem 34: 215-225.
4. Ein-Dor L, Kela I, Getz G, Givol D, Domany E (2005) Outcome signature genes in breast cancer: is there a unique set? Bioinformatics 21: 171-178.
5. Kalousis A, Prados J, Hilario M (2007) Stability of feature selection algorithms: a study on high-dimensional spaces. Knowledge and Information Systems 12: 95-116.
6. Duda R, Hart P, Stork D (2001) Pattern Classification and Scene Analysis.
7. Zhang X, Lu X, Shi Q, Xu XQ, Leung HC, et al. (2006) Recursive SVM feature selection and sample classification for mass-spectrometry and microarray data. BMC Bioinformatics 7: 197.
8. Eriksson L JE, Kettaneh-Wold N, Trygg J, Wikström C, Wold S (2006) Multi- and megavariate data analysis. Basic Principles and Applications.
9. Lindgren F, Hansen B (1996) Model validation by permutation tests Applications to variable selection. Journal of Chemometrics 10: 521-532.
10. Golland P, Liang F, Murkherjce S, Panchenko D (2000) Permutation tests for classification. Journal of Machine Learning Research.
11. Pesarin F, Salmaso L (2010) Permutation tests for complex data: Theory, applications and software.
12. Fisher RA (1937) The design of experiments.
